# Distinct Roles of Hemocytes at Different Stages of Infection by Dengue and Zika Viruses in *Aedes aegypti* Mosquitoes

**DOI:** 10.3389/fimmu.2021.660873

**Published:** 2021-05-13

**Authors:** Thiago H. J. F. Leite, Álvaro G. A. Ferreira, Jean-Luc Imler, João T. Marques

**Affiliations:** ^1^ Department of Biochemistry and Immunology, Instituto de Ciências Biológicas, Universidade Federal de Minas Gerais, Belo Horizonte, Brazil; ^2^ Mosquitos Vetores: Endossimbiontes e Interação Patógeno-Vetor, Instituto René Rachou – Fiocruz, Belo Horizonte, Brazil; ^3^ Université de Strasbourg, CNRS UPR9022, Inserm U1257, Strasbourg, France

**Keywords:** Zika virus, dengue virus, cellular immunity, macrophage-like cells, *Aedes aegypti*, vector mosquitoes, hemocytes

## Abstract

*Aedes aegypti* mosquitoes are vectors for arboviruses of medical importance such as dengue (DENV) and Zika (ZIKV) viruses. Different innate immune pathways contribute to the control of arboviruses in the mosquito vector including RNA interference, Toll and Jak-STAT pathways. However, the role of cellular responses mediated by circulating macrophage-like cells known as hemocytes remains unclear. Here we show that hemocytes are recruited to the midgut of *Ae. aegypti* mosquitoes in response to DENV or ZIKV. Blockade of the phagocytic function of hemocytes using latex beads induced increased accumulation of hemocytes in the midgut and a reduction in virus infection levels in this organ. In contrast, inhibition of phagocytosis by hemocytes led to increased systemic dissemination and replication of DENV and ZIKV. Hence, our work reveals a dual role for hemocytes in *Ae. aegypti* mosquitoes, whereby phagocytosis is not required to control viral infection in the midgut but is essential to restrict systemic dissemination. Further understanding of the mechanism behind this duality could help the design of vector-based strategies to prevent transmission of arboviruses.

## Introduction

*Aedes aegypti* mosquitoes are vectors for a wide variety of arthropod-borne viruses (arboviruses) (1). How these mosquitoes recognize and respond to viral infection is a central question that directly affects their vector competence. The understanding of antiviral responses in insects has greatly benefited from work in the fruit fly *Drosophila melanogaster* (2). Work in this model organism has identified many important antiviral defense mechanisms such as RNA interference (RNAi), Jak-STAT and STING (3–12). Later work in mosquitoes has shown that RNAi and Jak-STAT are important for the control of arbovirus infections (13–18). Interestingly, despite being widely conserved throughout evolution, STING has been lost in mosquitoes (19).

In addition to these well-known innate immunity pathways, the *Drosophila* model has also highlighted the role of circulating macrophage-like cells, referred to as hemocytes, in the control of viral infection (20–22). Cellular immunity in insects includes phagocytosis of foreign bodies, nodulation, wound healing and the encapsulation of pathogens (23–27). Hemocytes can be freely circulating in the insect hemolymph or associated with tissues but these populations seem to be highly dynamic and interchangeable (28). Hemocytes are often recruited to infected tissues, which increases the chances of coming into contact with the pathogen to be cleared by phagocytosis (28, 29). A good example of hemocyte recruitment during an infection is in the case of *Plasmodium*, the malaria parasite. Invasion of the midgut of *Anopheles* mosquitoes by *Plasmodium* ookinetes promotes hemocyte recruitment and release of components of the mosquito complement system, promoting pathogen elimination (30–34). Despite the importance of hemocytes for the clearance of bacteria and *Plasmodium* in mosquitoes, little is known about their role during viral infections, particularly arboviruses such as dengue (DENV) and Zika (ZIKV) viruses. DENV and ZIKV belong to the *Flaviviridae* family and, together with the alphavirus chikungunya virus (CHIKV) are among the most important arboviruses transmitted by *Ae. aegypti* mosquitoes causing infections worldwide (1). Similar to the malaria parasite, arboviruses are acquired orally during blood feeding by mosquitoes, and the gut represents a physical barrier that hinders the passage of the viral particles to the mosquito hemocele (35). Reaching the hemocele is a necessary step for the virus to spread systemically and reach the salivary glands where it can be transmitted to a vertebrate host (21–23). During systemic infection, several tissues may host viral replication, including hemocytes themselves, but it is unclear how they contribute to amplification of the virus (36–38). Despite this increasing knowledge about the functions of hemocytes in mosquitoes, the role of cellular immunity in the antiviral defense remains largely unknown.

In this work, we investigated the involvement of hemocytes in the control of DENV and ZIKV in *Ae. aegypti* mosquitoes. Our results suggest a complex role for hemocytes. We show that hemocytes were recruited to the midgut in response to the presence of the virus but, once there, their phagocytic activity seems to facilitate viral replication although other functions my play a role in the antiviral defense. In contrast, during the systemic phase of the infection, inhibition of phagocytosis by hemocytes led to increased viral infection pointing to a more traditional role in antiviral immunity. Together our results indicate that hemocytes have dual roles in the control of arboviruses in *Ae. aegypti* mosquitoes depending on tissue affected and the stage of the infection in the vector.

## Materials and Methods

### Indirect Immunofluorescence Assays

Mosquitoes were anaesthetized on ice and then were inject with 250 nanoliters of 20% paraformaldehyde for hemocyte fixation in midgut basal lamina. After 20 minutes, midguts were dissected in 4% paraformaldehyde diluted in phosphate-buffered saline (PBS) (13 mM NaCl, 0.7 mM Na2HPO4, 1 mM NaH2PO4 at pH 7.2) (PBS). The remaining midguts were fixed in the same solution for 20 minutes, and then washed three times in PBS and then incubated with blocking solution PBSBT (1× PBS + 1% BSA + 0.1% Triton X-100) for 15 minutes at room temperature. Samples were then incubated overnight with 4G2 monoclonal antibody for Flavivirus E protein (ATCC: HB-112, used at 1:50 in PBST) at 4°C. Midguts were washed three times with PBSBT (5 min each) and incubated for 2 h with constant rocking at 25°C with goat anti-mouse IgG antibody (Invitrogen). Midguts were washed three times with PBST (5 min each) and incubated for 15 min with DAPI (Molecular Probes, 1:500), and phalloidin-rhodamine (Molecular Probes, 1:500). Then the midguts were washed in PBS and placed onto slides. Images were obtained with an LSM 880 microscope (Zeiss).

### Mosquito Perfusion to Obtain Circulating Hemocytes

Circulating hemocytes were obtained by perfusion of adult mosquitoes as described (39) with modifications. Briefly, mosquitoes were injected with 1 uL of anticoagulant buffer solution (70% PBS 1x (pH 7.0) + 30% citrate buffer (98 mM NaOH, 186 mM NaCl, 1.7 mM EDTA and 41 mM citric acid, buffer pH 4.5) and were incubated on a petri dish on ice for 10 min to let hemocytes dissociate from tissues. The last two segments of abdomen were cut to create an opening, which was positioned onto a microscope slide. Each individual mosquito was positioned vertically and then injected with 3 uL of the same anticoagulant buffer solution in the lateral side of torax using a microinjector (Nanoject III). The injection pressure forced the diluted hemolymph to exit the opening made in the final portion of the abdomen and onto the microscope slide. The hemolymph was incubated at room temperature for 20 min in order to let the hemocytes adhere to the slides. Hemocytes were fixed in 4% paraformaldehyde for 20 min, washed three times in PBS and then incubated with blocking solution PBSBT (1× PBS + 1% BSA + 0.1% Triton X-100) for 15 minutes at room temperature. Slides were incubated for 15 min with DAPI (Molecular Probes, 1:500) and phalloidin-rhodamine (Molecular Probes, 1:40), followed by 3 washes in PBS. Cells were visualized in a fluorescence microscope for counting. To visualize infected hemocytes, the 4G2 monoclonal antibody against Flavivirus E protein was used.

### Hemocyte Labeling *In Vivo*

For *in vivo* hemocyte staining we used Vybrant™ CM-DiI Cell-Labeling Solution (Invitrogen™) essentially as described (29). Briefly, female mosquitoes were placed on petri dish on ice and injected with 150 nanoliters containing 100 μM CM-DiI, freshly prepared in sterile water, after blood or sugar meal at specific time points. Injections were done using a nano-injector Nanoject III (Drummond Scientific Company). After injections, mosquitoes are placed on cages at 28°C until specific time points for midguts dissections.

### Quantification of the Infection Area in the Midgut

Area measurements and hemocyte counting were performed using ImageJ v1.53c (https://imagej.nih.gov/ij/). All images were acquired under identical conditions, digitized, converted to RGB image and stored in an uncompressed tagged image file format (.tiff). Infection area computing was performed using ImageJ. The following steps were performed for all images to quantify the area of infection in the midgut, as shown in **Supplementary Figure 1**: step 1, color-deconvolution was used to isolate red, green and blue spectra and select the image corresponding to virus infection staining; step 2, a projection final image was generated using all acquired series of z-stack confocal images using the tool “Image > Stacks > Z-project function”; step 3, the projection image was processed into 8 bits image type; step 4, the midgut outline was delimited; step 5, the area outside of midgut delimitation was erased by using the “clear outside” function; steps 6 and 7, optical density was assessed by setting a threshold using the “threshold tool”, and a maximum threshold was set; steps 8 and 9, the function “Measure” in the ‘Analyze’ tool menu was used to calculate the optical density and compute the midgut infection area.

### Quantification of Hemocytes in the Midgut

Hemocyte numbers were quantified in the confocal microscopy images of midguts. The following steps were performed using ImageJ for all images: step 1, color-deconvolution was used to isolate red, green and blue spectra and select the images corresponding to hemocytes cell tracker and DNA staining; step 2, for each color, a projection final image was generated using all acquired series of z-stack confocal images using the tool “Image > Stacks > Z-project function”; step 3, the projection image was processed into 8 bits image type; step 4, the number of hemocytes was then counted using the ITCN (Image-based Tool for Counting Nuclei); step 5, for all hemocytes automatically identified in the hemocytes cell tracker color we additionally confirmed the presence of nuclei using the DNA staining image and reject the counts that do not presented a nucleus.

### Inhibition of Phagocytosis by Injection of Latex Beads

To block the phagocytic activity of hemocytes in mosquitoes, we adapted protocols previously used for *Drosophila* (20). Adult mosquitoes were injected with 69 nanoliters of latex microspheres (CML Latex Beads, 4% w/v, 0.3 µm, ThermoFisher). Latex beads were washed and resuspended at a 2X concentration in PBS before injections. In order to quantify the inhibition of phagocytosis, we first injected regular latex beads followed by injection of red fluorescent beads (FluoSpheres ™ Carboxylate-Modified Microspheres, 0.2 μm, dark red fluorescent (660/680), 2% solids, ThermoFisher) two days later. Perfusions were done 4 and 8 days after the first injection and the total number of hemocytes was counted as well as the percentage of cells with red beads.

### RT-qPCR

Total RNA (200 ng) extracted from individual insects or individual tissues was reverse transcribed using Moloney murine leukaemia virus reverse transcriptase. cDNA was subjected to quantitative PCR (qPCR) using the kit Power SYBR Green Master Mix (Applied Biosystems), following the manufacturer’s instructions. Primers used for quantitative PCR (qPCR) were as follows: RPL32 (forward, 5´-ACTTCTTCGTCCGCTTCTTG-3´; reverse, 5´-AGCCGCGTGTTGTACTCTG-3´), DENV1 (forward, 5´-TCGGAAGCTTGCTTAACGTAG-3´; reverse, 5´ TCCGTTGGTTGTTCATCAGA-3´), ZIKV (forward, 5´-TCAAACGAATGGCAGTCAGTG-3´; reverse, 5´-GCTTGTTGAAGTGGTGGGAG-3´) as previously described (14).

### Mosquito Rearing and Infections

All experiments were carried out using *Ae. aegypti* Bangkok strain. Mosquitoes were maintained in an incubator at 28°C and 70–80% relative humidity, in a 12:12 h light:dark photoperiod, and with 10% sucrose solution ad libitum. For mosquito infections, we used previously described models for flavivirus infections using mice or artificial membrane feeding. Isolates of DENV4 (H241 strain), DENV1 (MV09) and ZIKV (PE243/2015) were previously described (14). As a mouse model, we utilized DENV1 and ZIKV infection of interferon alpha/beta and gamma receptor-deficient (AG129) animals (14). Mice were injected intraperitoneally with 10^6^ pfu/mL of virus. Infected mice were anaesthetized at 3 days post injection (peak of viraemia) using ketamine/xylazine (80/8 mg kg^−1^) and placed on top of the netting-covered containers with 5- to 6-day-old adult mosquito females. For infections by artificial membrane feeding, 5-6 day old adult females were starved for 24h and fed with a mixture of blood and virus supernatant containing 10^7^ pfu/mL of DENV4 or 10^6^ pfu/mL of ZIKV utilizing a glass artificial feeding system covered with pig intestine membrane, essentially as described (14). Mosquitoes were allowed to feed for 1 h. After blood feeding, fully engorged females were selected and harvested individually for midgut dissection at different time points. For direct systemic infections by intrathoracic injections, mosquitoes were anaesthetized with CO2 and kept on ice during the whole procedure. 4-day-old females were intrathoracically injected with 69 nL of L15 media containing virus (5 or 50 pfu), using a nano-injector Nanoject III (Drummond Scientific Company). Mosquitoes were harvested at different days post injection for RNA extraction. Tissues or mosquitoes were ground in TRIzol (Invitrogen) using glass beads. Total RNA was extracted from individual mosquitoes or individual tissues according to the manufacturer’s protocol.

## Results

### DENV and ZIKV Trigger Accumulation of Hemocytes in the Mosquito Midgut

**Figure 1:**
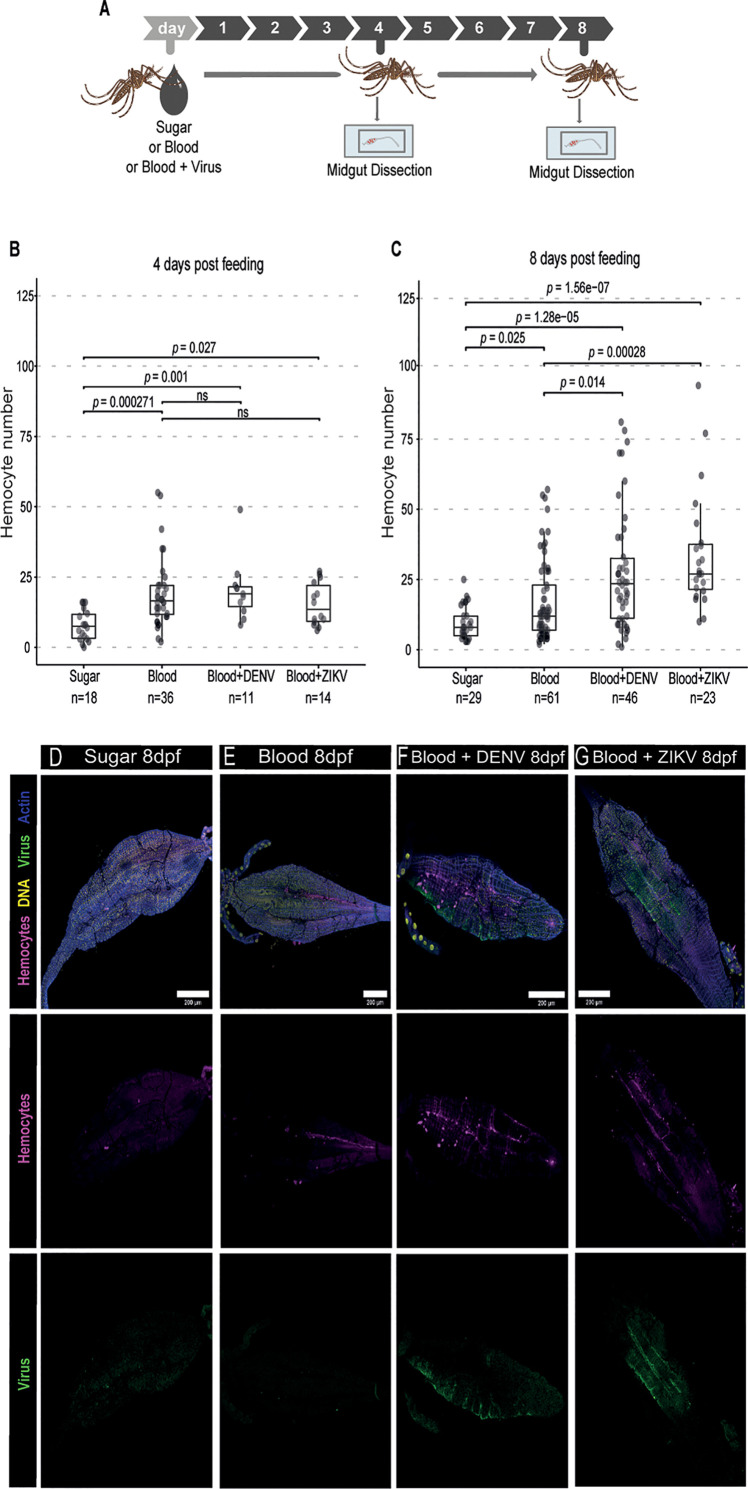
Hemocyte accumulation in the midgut of *Ae. aegypti* mosquitoes in response to DENV and ZIKV. **(A)** Mosquitoes fed with sugar, blood or blood and virus were dissected at different times and their midgut was analyzed by confocal microscopy. Virus-infected mice were used as a source of blood. **(B, C)** Quantification of the number of midgut-associated hemocytes between mosquitoes fed with sugar, blood or blood and virus at 4 **(B)** and 8 **(C)** days post feeding. DENV and ZIKV were analyzed together. 2 independent experiments for each virus were pooled. Each dot represents an individual midgut. Total number of midguts tested is indicated below each box plot. Upper, middle and lower bars in the boxplot represent the 75th percentile, the median and the 25th percentile, respectively. Statistical analyses were performed using the Kruskal-Wallis test followed by Dunn’s test to correct for multiple comparisons. ns, non-significant. **(D–G)** Representative confocal microscopy images of mosquito midguts showing CM-DiL stained hemocytes in magenta, DNA in yellow, viral E proteins in green and actin in blue. Midguts from mosquitoes fed with sugar **(D)**, blood **(E)**, blood + DENV **(F)** and blood + ZIKV **(G)** are shown at 8 days post feeding.

Hemocytes play an important role in mosquito immunity but their function in the antiviral response against arboviruses remains unclear. Here, we first analyzed whether hemocytes would respond to the presence of arboviruses, DENV and ZIKV, in the blood meal (**Figure 1A**). Others have observed that blood feeding induces an increase in the numbers of hemocytes in mosquitoes (40, 41). Here we observed that there is also an increase in the number of hemocytes associated with the midgut compared to mosquitoes that were kept on sugar at 4 and 8 days post feeding (**Figures 1B, C**). At the earlier time point, there was no significant difference between the number of hemocytes associated with the midgut of mosquitoes fed with blood or blood and virus (**Figure 1B**). However, at 8 days post feeding, numbers of midgut-associated hemocytes were significantly higher in the presence of DENV or ZIKV when compared to a control blood meal (**Figure 1C**). Notably, these hemocytes do not seem to be recruited to sites of viral replication. We observed that hemocytes were often found dispersed throughout the midgut and not necessarily concentrated around regions with staining of the viral E protein as an indication of infection (**Figures 1D–G**). These results suggest that the presence of virus particles in the blood meal increases the number of hemocytes associated to midgut possibly by providing signals for increased recruitment or longer retention of these cells in the organ. The delayed effect at later times post infection also suggests that the accumulation of hemocytes may require prolonged stimuli.

### Phagocytosis by Hemocytes Does Not Contribute to the Control of DENV and ZIKV in the Midgut

**Figure 2:**
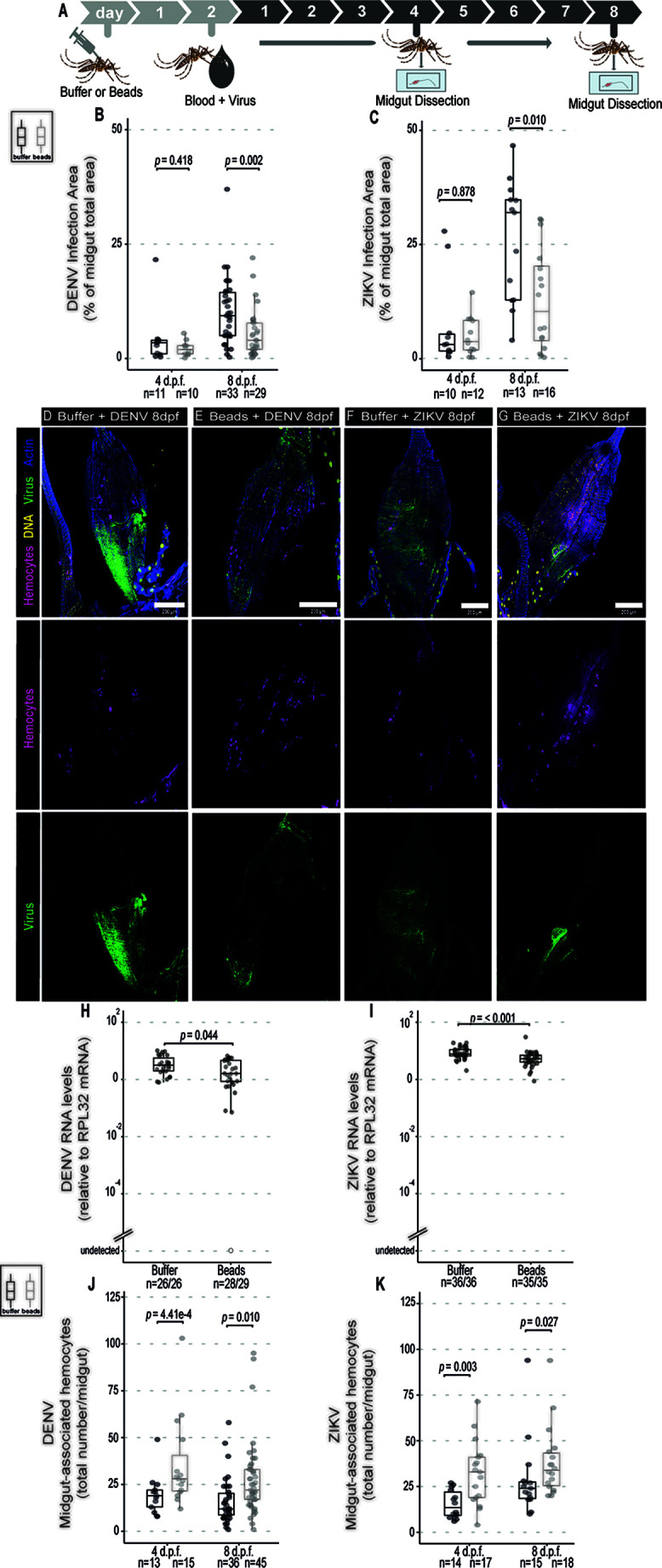
Phagocytosis by hemocytes is not required to control DENV and ZIKV infection in the midgut of *Ae. aegypti* mosquitoes. **(A)** Mosquitoes injected with latex beads were fed 2 days later with blood + virus and dissected at different times to be analyzed. Virus-infected mice were used as a source of blood. **(B, C)** Percentage of total midgut infection area that shows staining for the viral protein at 4 and 8 days post infection was determined by immunofluorescence. Total number of midguts tested is indicated below each box plot. DENV **(B)** and ZIKV **(C)** were analyzed separately. 2 independent experiments for each virus were pooled. Each dot represents an individual midgut. **(D–G)** Representative confocal microscopy images of mosquito midguts showing CM-DiL stained hemocytes in magenta, DNA in yellow, viral E proteins in green and actin in blue. **(D, E)** Midguts from mosquitoes fed on blood + DENV. **(F, G)** Midguts from mosquitoes fed on blood + ZIKV. **(D, F)** Midguts from control mosquitoes injected with buffer; **(E, G)** Midguts from mosquitoes injected with latex beads. **(H)** DENV and **(I)** ZIKV RNA levels measured by RT-qPCR at 8 days post feeding. The number of positive midguts over the total tested is indicated below each boxplot. **(J, K)** Number of midgut-associated hemocytes in individual midguts from control and virus infected mosquitoes at 4 and 8 days post feeding. DENV **(J)** and ZIKV **(K)** were analyzed separately. Total number of midguts tested is indicated below each box plot. 2 independent experiments for each virus were pooled. **(B, C, H–K)** Each dot represents an individual midgut. Upper, middle and lower bars in the boxplot represent the 75th percentile, the median and the 25th percentile, respectively. Statistical analyses were performed using the Mann-Whitney-Wilcoxon test.

Increased numbers of hemocytes in the midgut in response to arboviruses in the blood meal suggests that these cells may play a role in the antiviral defense. Phagocytosis is a major function of hemocytes. Indeed, blocking phagocytosis by hemocytes or complete genetic ablation of these cells leads to decreased resistance to viruses in *Drosophila* (20–22). Here, we decided to use injection of latex beads into mosquitoes, which is often used as a strategy to over-load hemocytes and inhibit their phagocytic capacity (20, 21, 42). In our experiments, we observed that injection of beads seemed to decrease the number of circulating hemocytes in the mosquito but that was not significant (**Supplementary Figure 2A**). The number of hemocytes was estimated in a fraction of the hemolymph obtained by perfusion of mosquitoes with a low volume of buffer. Although this strategy recovered smaller numbers of hemocytes compared to other methods (39, 40), it still allowed us to compare numbers of cells between two conditions, which was our objective. Using the same strategy, we observed that phagocytosis by hemocytes was significantly inhibited by latex beads 2 days after their injection into *Ae. aegypti* mosquitoes (**Supplementary Figure 2B**). We next analyzed the effect of latex beads in mosquitoes that were given a blood meal containing DENV or ZIKV 2 days later, during the time when phagocytosis is inhibited (**Figure 2A**). Blocking phagocytosis did not affect significantly the area of infection by DENV or ZIKV in the midgut at 4 days post feeding (**Figures 2B, C**). In contrast, at 8 days post feeding, we observed that midgut of mosquitoes injected with latex beads had a significantly decreased area of infection by DENV and ZIKV compared to controls (**Figures 2B–G**). Importantly, injection of latex beads did not significantly change the total size of the midgut at the same time point but affected the absolute infection area suggesting that the kinetics of viral replication itself was affected (**Supplementary Figure 3**). At 4 days post feeding, injection of beads caused a reduction in viral RNA levels in DENV and ZIKV infected mosquitoes, although it was only significant for the latter (**Supplementary Figure 4**). At 8 days post feeding, DENV and ZIKV RNA levels were also significantly decreased in midguts from mosquitoes injected with latex beads compared to controls (**Figures 2H, I**). These results suggest that blocking the phagocytic activity of hemocytes using latex beads led to decreased viral replication in the midgut of mosquitoes. Notably, we consistently observed that latex beads increased the number of midgut-associated hemocytes in sugar and blood fed mosquitoes, independent of virus infection (**Supplementary Figure 5**). Latex beads also increased numbers of hemocytes in the midgut of DENV and ZIKV infected mosquitoes at 4 and 8 days post feeding (**Figures 2J, K**). During viral infection, latex beads had a less striking effect on hemocyte numbers at later time points since infection itself led to accumulation of hemocytes in the midgut (**Figure 1C**). This increased accumulation of hemocytes in the midgut induced by latex beads preceded the reduction in viral levels. Thus, we cannot rule out that increased accumulation of hemocytes in the midgut induced by beads is helping control viral infection but this would have to occur independently of their phagocytic activity.

### Phagocytosis by Hemocytes Is Required for Systemic Control of DENV and ZIKV

**Figure 3:**
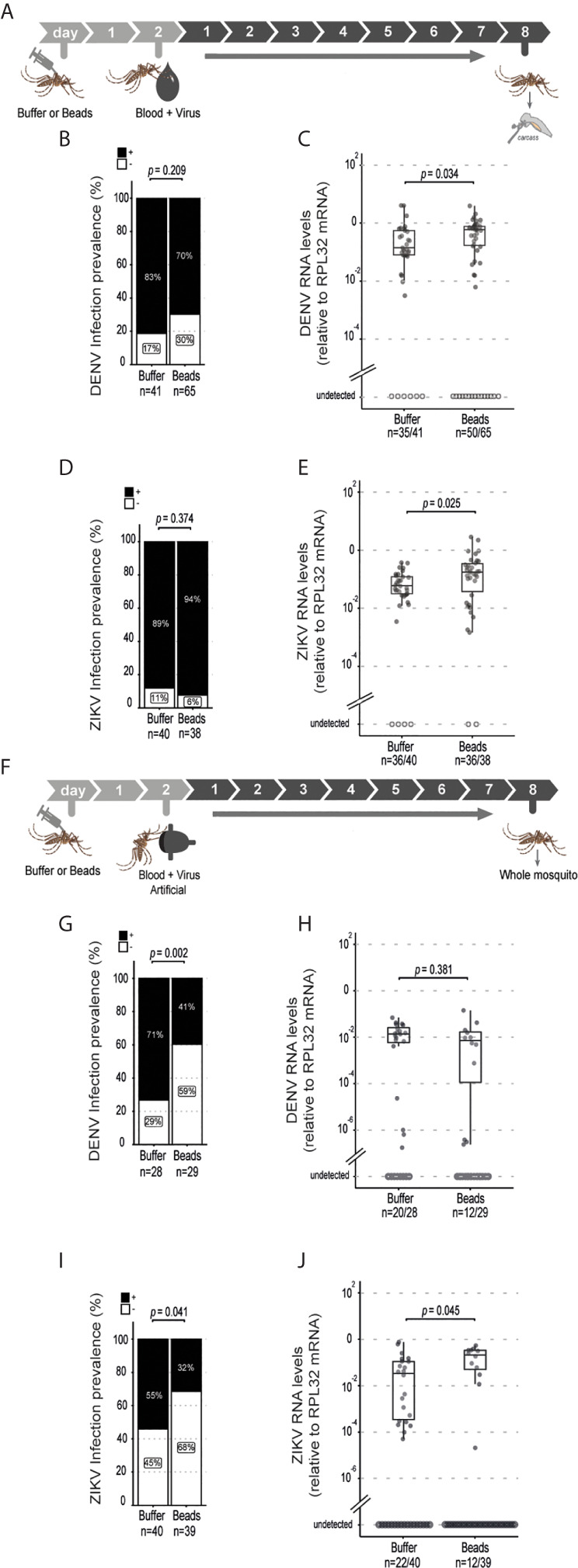
Systemic dissemination of ZIKV and DENV infection is controlled by hemocyte phagocytosis. **(A)** Mosquitoes injected with latex beads were fed 2 days later with blood and virus and dissected at 8 days post feeding to be analyzed. Virus-infected mice were used as a source of blood. **(B, C)** Prevalence of DENV **(B)** and ZIKV **(C)** infection in the mosquito carcass at 8 days post feeding. The number of positive mosquitoes over the total tested is indicated below each column. One representative experiment is shown. This experiment was repeated 3 times for DENV and once for ZIKV. Statistical analyses were performed using two-tailed Fishers exact test. **(D, E)** Viral RNA levels at 8 days post feeding for DENV **(D)** and ZIKV **(E)**. One representative experiment is shown. This experiment was repeated 3 times for DENV and once for ZIKV. Each dot represents an individual mosquito. **(F)** Mosquitoes injected with latex beads were given an artificial blood meal with virus 2 days later and analyzed at 8 days post feeding. **(G, H)** Prevalence of DENV **(G)** and ZIKV **(H)** infection in mosquitoes injected with buffer or beads. The number of positive mosquitoes over the total tested is indicated below each column. One representative experiment is shown. This experiment was repeated twice for DENV and once for ZIKV. Statistical analyses were performed using two-tailed Fishers exact test. **(I, J)** DENV **(I)** and ZIKV **(J)** RNA levels in mosquitoes injected with buffer or beads. Each dot represents an individual mosquito. The number of positive mosquitoes over the total tested is indicated below each boxplot. One representative experiment is shown. This experiment was repeated twice for DENV and once for ZIKV. **(D, E, I, J)** Upper, middle and lower bars in the boxplot represent the 75th percentile, the median and the 25th percentile, respectively. Statistical analyses were performed using the Mann-Whitney-Wilcoxon test.

**Figure 4:**
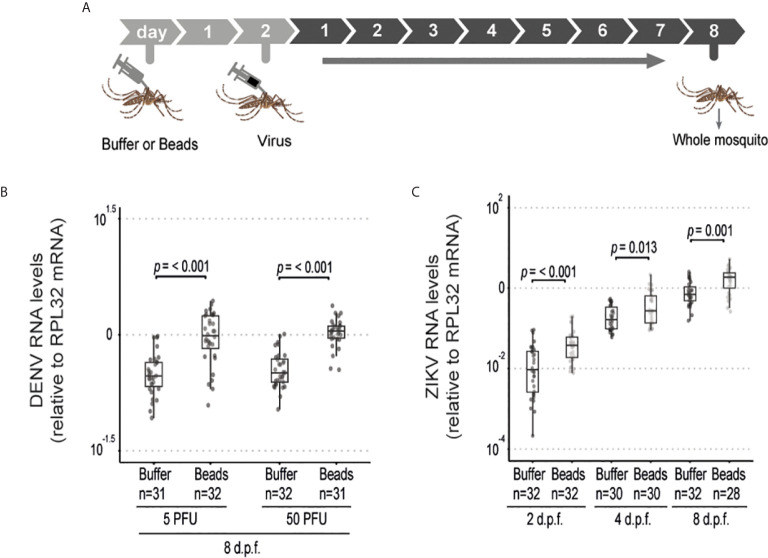
Phagocytosis by hemocyte is required to inhibit systemic replication of ZIKV and DENV. **(A)** Mosquitoes injected with latex beads were subsequently injected with virus 2 days later and samples were analyzed at different time points. **(B)** Viral RNA levels in mosquitoes injected with 5 or 50 PFU of DENV at 8 days post injection. **(C)** Viral RNA levels in mosquitoes injected with 5 PFU of ZIKV at 2, 4 and 8 days post injection. One representative experiment is shown. This experiment was repeated twice for DENV and once for ZIKV. **(B, C)** Each dot represents an individual mosquito. The number of positive mosquitoes over the total tested is indicated above each boxplot. Upper, middle and lower bars in the boxplot represent the 75th percentile, the median and the 25th percentile, respectively. Statistical analyses were performed using the Mann-Whitney-Wilcoxon test.

The above results suggest that phagocytosis is not involved in the control of viral infection in the midgut of *Aedes* mosquitoes. This contrasts with the well-known roles of phagocytosis by hemocytes in insect immunity especially in the antiviral defense of *Drosophila*. However, these cells have also been shown to host replication of arboviruses such as DENV, Sindbis and O’nyong’nyong virus, which could help explain a proviral function (36–38). We confirmed that hemocytes could be directly infected by ZIKV as indicated staining for the viral E protein (**Supplementary Figure 6**). Thus, phagocytosis of viral particles by hemocytes could help promote viral replication in mosquitoes. In order to look further into this possibility, we analyzed dissemination of DENV and ZIKV infection from the midgut to the carcass in mosquitoes injected with latex beads (**Figure 3A**). Although the midgut infection rate was significantly reduced when phagocytosis was inhibited (**Figures 2B–I**), this did not significantly affect the prevalence of mosquitoes with disseminated infection (**Figures 3B, C**). Nevertheless, we observed a significant increase in viral RNA levels in the carcass of mosquitoes infected with DENV and ZIKV when phagocytosis by hemocytes was inhibited (**Figures 3D, E**). Here we note that mosquitoes fed on viremic mice show over 80% prevalence of infection. Therefore, to further analyze a possible effect of latex beads on the dissemination, we decided to analyze a model of artificial blood feeding where virus concentrations could be more easily controlled to reach closer to 50% prevalence (**Figure 3F**). In this model, injection of beads into mosquitoes prior to blood feeding containing DENV or ZIKV lead to a significant decrease in the prevalence of infection (**Figures 3G, H**). At the same time, viral loads were not significantly different for DENV and were increased in ZIKV infected individuals when phagocytosis was inhibited (**Figures 3I, J**). This reinforces the idea that blocking phagocytosis by hemocytes leads to decreased midgut replication that results in lower systemic dissemination. In order to bypass the midgut and directly analyze the role of hemocytes during systemic viral replication, we used a model of intrathoracic injection of the virus (**Figure 4A**). Consistent with results using the oral infection models, we observed that inhibition of the phagocytic activity of hemocytes led to a clear increase in systemic viral replication after injection of DENV and ZIKV (**Figures 4B, C**). This effect was highly significant and did not depend on the dose of virus used or the kinetics of infection. Together, our data indicate that phagocytosis by hemocytes is essential to control systemic viral replication, which is consistent with their important roles in cellular immunity.

## Discussion

Here we have studied the role of phagocytosis by insect macrophage-like cells in the control of DENV and ZIKV in *Ae. aegypti* mosquitoes. These macrophage-like cells, known as hemocytes, are important components of the mosquito immune system (25). We and others have previously shown that phagocytosis by these cells plays an important function in the antiviral defense of *Drosophila* (20–22) but their role during viral infections in mosquitoes remain unclear.

Our results show that hemocytes accumulate in the midgut of *Ae. aegypti* mosquitoes in response to the presence of ZIKV and DENV in the blood meal. Interestingly, increased numbers of hemocytes in the midgut are not observed at 4 days post infection but only later at 8 days, suggesting it either requires continuous stimulation or is triggered only after certain levels of viral replication. Since the infection did not significantly change the number of circulating hemocytes, these results suggest that these cells were recruited or retained more efficiently in the midgut. Increased numbers of hemocytes in the midgut suggests an important role for these cells in the response to viral infection. However, our results were less clear regarding their possible function in the midgut. We observed that blocking phagocytosis by hemocytes using latex beads led to decreased virus replication in the midgut after 8 days post infection when these cells accumulate significantly. We show that phagocytosis is inhibited at 2 days post injection of latex beads at the time of viral infection in the midgut. Although it is unclear how long this inhibition lasts, these results suggest that phagocytosis by hemocytes has a proviral function during the early stages of DENV and ZIKV infection in the midgut. However, when phagocytosis was blocked by latex beads, we also observed increased numbers of hemocytes in the midgut of mosquitoes as early as 4 days post infection. This effect was independent of viral replication or blood feeding and could be related to lower motility of hemocytes after phagocytosis since we do observe a tendency of decreased numbers of circulating hemocytes after injection of beads. Nevertheless, increased numbers of hemocytes in the midgut precede and could be responsible for the inhibition of viral replication independent of phagocytosis. Phagocytosis of latex beads does not seem to prime hemocytes for wound healing in *Drosophila* (26), which would suggest that these hemocytes in the mosquito midgut are not activated but rather inert. Our current data do not allow us to rule out that there are other antiviral functions by hemocytes triggered by latex beads (e.g. production of antiviral cytokines) but it is clear that phagocytosis is not required to control viral infection in the midgut of mosquitoes. Paradoxically, when the infection disseminates from the midgut, phagocytosis by hemocytes has an important role controlling systemic viral replication. Together, our data point to a dual role for phagocytosis by hemocytes in the antiviral response of *Ae. aegypti* mosquitoes against DENV and ZIKV. Phagocytosis does not affect virus replication in the midgut but is essential to control systemic infection. Notably, work by our groups and others have pointed to important differences in the requirements to control viral replication in the midgut compared to systemic infection in mosquitoes (14, 36). For example, RNA interference plays a major role during systemic infection but has little contribution to the control of viral replication in the midgut (14). Similarly, apoptosis, which is coupled to phagocytosis by hemocytes to restrict viral infection in *Drosophila* (20, 21), may not be efficiently induced in response to virus infection in the midgut epithelium of *Aedes* mosquitoes

The reason for contrasting roles of hemocytes during infection of the midgut compared to systemic dissemination of DENV and ZIKV suggests a complex scenario. It is possible that hemocytes might carry out immune functions that have opposing impacts over viral infection whether in the midgut or systemically. For example, phagocytosis might be important to clear viruses from the circulation but, in the midgut, could help virus dissemination. However, recent single cell analyses have indicated that hemocytes are composed of many subgroups that likely have distinct functions in immunity (43–46). Based on these data, it is possible that epithelial and systemic responses to viral infections mobilize different subtypes of hemocytes. Upon blood feeding, there is extensive damage to the basal lamina of the midgut and this is further exacerbated by infection by chikungunya virus (47, 48). ZIKV causes similar damage to the basal lamina (49) and that is likely true for other arboviruses. Damage to the basal lamina presumably leads to the recruitment of certain subtypes of hemocytes to these damaged regions with high concentration of the virus (47, 49). It is possible that hemocytes that are recruited to repair this damage become infected and help amplify local viral replication. Alternatively, these hemocytes could promote enterocyte survival or intestinal stem cell proliferation (50–53) and thus favor viral replication in the midgut. In contrast, during systemic dissemination of ZIKV and DENV, other subtypes of hemocytes would then play a more classical antiviral role by clearing particles and infected cells (20). Hemocytes may also participate in a systemic antiviral RNA interference of mosquitoes, as proposed in Drosophila (22), and this may not be functional in the midgut. Notably, recent work in *Anopheles* mosquitoes has suggested that subtypes of hemocytes may have different roles during specific stages of *Plasmodium* infection (34). These are pressing questions that we are currently investigating to elucidate the mechanism by which hemocytes contribute to the antiviral defense. Alternative methods for hemocyte depletion (34, 54) or genetic approaches to ablate or interfere with cell function in mosquitoes will be important tools for the field going forward. These studies will help understand how vector mosquitoes recognize and fight viral infections that could lead to novel strategies to control transmission of arboviruses.

## Data Availability Statement

The original contributions presented in the study are included in the article/**Supplementary Material**. Further inquiries can be directed to the corresponding author.

## Ethics Statement

The animal study was reviewed and approved by Comissão de Ética no uso de animais - CEUA da UFMG (337/2016).

## Author Contributions

TL, AF, J-LI, and JM designed the experiments. TL and AF carried out the experiments and statistical analysis. TL, AF, J-LI, and JTM analyzed results. TL, AF, J-LI, and JM wrote the manuscript. All authors contributed to the article and approved the submitted version.

## Funding

This work was funded by Conselho Nacional de Desenvolvimento Científico e Tecnológico (CNPq), Fundação de Amparo a Pesquisa do Estado de Minas Gerais (FAPEMIG), Rede Mineira de Imunobiologicos (grant # REDE-00140-16), Instituto Nacional de Ciência e Tecnologia de Vacinas (INCTV), the Institute for Advanced Studies of the University of Strasbourg (USIAS fellowship 2019) and the Investissement d’Avenir Programs (ANR-10-LABX-0036 and ANR-11-EQPX-0022). This study was financed in part by the Coordenação de Aperfeiçoamento de Pessoal de Nível Superior - Brasil (CAPES) - Finance Code 001.

## Conflict of Interest

The authors declare that the research was conducted in the absence of any commercial or financial relationships that could be construed as a potential conflict of interest.
