# Supplementary material for: Distinct Roles of Hemocytes at Different Stages of Infection by Dengue and Zika Viruses in Aedes aegypti Mosquitoes
Source: Front Immunol. 2021 May 13;12:660873. doi: 10.3389/fimmu.2021.660873 (PMC8169962; doi:10.3389/fimmu.2021.660873)

Distinct roles of hemocytes at different stages of infection by Dengue and Zika viruses in *Aedes aegypti* mosquitoes

Thiago H. J. F. Leite[1,#], Álvaro G. A. Ferreira[1,2,#], Jean-Luc Imler[3], João T. Marques[1,3*]

[1]Department of Biochemistry and Immunology, Instituto de Ciências Biológicas, Universidade Federal de Minas Gerais, Belo Horizonte, Brazil, CEP 31270-901

[2]Mosquitos Vetores: Endossimbiontes e Interação Patógeno-Vetor, Instituto René Rachou – Fiocruz, Belo Horizonte, Brazil

[3]Université de Strasbourg, CNRS UPR9022, Inserm U1257, 67084 Strasbourg, France

# These authors contributed equally to this work

*** Correspondence:**

João T. Marques, email: jtm@ufmg.br

Keywords: hemocytes, macrophage-like cells, cellular immunity, vector mosquitoes, *Aedes aegypti*, dengue virus, Zika virus

Running title: Mosquito hemocytes and flavivirus infections

Supplementary Figure Legends

Supplementary Figure 1 – Experimental design for the quantification of the midgut infection area. Infection area computing was performed using ImageJ following 9 steps as detailed in the figure: Step 1, color-deconvolution was used to isolate red, green and blue spectra and select the image corresponding to virus infection staining. Step 2, since a series of z-stack confocal images were acquired, a projection final image was generated using all z-series, “Image > Stacks > Z-project function”. Step 3, the projection image was processed into 8 bits image type. Step 4, the midgut outline was delimited. Step 5, the area outside of midgut delimitation was erased by using the “clear outside” function. Steps 6 and 7, optical density was assessed by setting a “threshold” using the “threshold tool”, and a maximum threshold was set. Steps 8 and 9, the function “Measure” in the ‘Analyze’ tool menu was used to calculate the optical density and compute the midgut infection area.

Supplementary Figure 2 – Latex beads do not significantly affect numbers of hemocytes but efficiently block phagocytosis. (A) Mosquitoes injected with latex beads were blood fed two days later. Mosquitoes kept fed on sugar during the whole period were used as controls. Hemocytes were counted after perfusion of mosquitoes at 4 and 8 days post feeding. Total number of mosquitoes is indicated above each box plot. Each dot represents an individual mosquito. Upper, middle and lower bars in the boxplot represent the 75th percentile, the median and the 25th percentile, respectively. Statistical analyses were performed using the Mann-Whitney-Wilcoxon test. (B) Mosquitoes were injected with latex beads or buffer and two days later again injected with fluorescent beads. Hemocytes were counted after perfusion of mosquitoes and the number of cells with and without fluorescent beads was determined at 4 and 8 days post injection. The percentage of cells with red fluorescent beads was plotted as the phagocytic index. The number of mosquitoes analyzed is indicated above each boxplot. Upper, middle and lower bars in the boxplot represent the 75th percentile, the median and the 25th percentile, respectively. Statistical analyses were performed using a general linear model with the ratios of phagocytic activity as response variable and explanatory factors of buffer vs beads, days post feeding, experiment replicate and all second-order interactions. The model used simplified by progressively removing terms. Terms were only removed if it did not cause a significant drop in the proportion of the total deviance explained. In the final models residuals were checked for normality and for the presence of outliers with high leverage.

Supplementary Figure 3 – Latex beads affect the absolute area of infection but not the size of the mosquito midgut. (A-D) Total midgut size in ZIKV (A, B) or DENV (C, D) infected mosquitos that were injected with latex beads compared to controls at 4 (A, C) and 8 (B, D) days post feeding. (E-H) Absolute area of infection by ZIKV (E, F) or DENV (G, H) in the midgut of mosquitoes injected with latex beads compared to controls at 4 (E, G) and 8 (F, H) days post feeding. Each dot represents an individual sample. The number of virus positive midguts over the total analyzed is indicated below each boxplot. Upper, middle and lower bars in the boxplot represent the 75th percentile, the median and the 25th percentile, respectively. Statistical analyses were performed using the Mann-Whitney-Wilcoxon test.

Supplementary Figure 4 – Inhibition of hemocytes marginally affects viral RNA levels of DENV and ZIKV in the midgut at 4 days post blood feeding. (A,B) Viral RNA levels at 4 days post infection with DENV (A) and ZIKV (B) in the midgut of mosquitoes injected with latex beads compared to controls. Each dot represents an individual midgut. The number of positive midguts over the total tested is indicated below each boxplot. Upper, middle and lower bars in the boxplot represent the 75th percentile, the median and the 25th percentile, respectively. Statistical analyses were performed using the Mann-Whitney-Wilcoxon test.

Supplementary Figure 5 – Latex beads increase the number of midgut associated hemocytes in the absence of virus infection. Latex beads were injected into mosquitoes that were kept on sugar (A) or blood fed 2 days after injection (B). Mosquitoes kept on sugar feeding were used as controls. The number of midgut associated hemocytes was determined in mosquitoes at 4 and 8 days post feeding. The number of midguts analyzed in indicated above each boxplot. Each dot represents an individual midgut. Upper, middle and lower bars in the boxplot represent the 75th percentile, the median and the 25th percentile, respectively. Statistical analyses were performed using the Mann-Whitney-Wilcoxon test.

Supplementary Figure 6 – Hemocytes are productively infected by ZIKV. Representative images of confocal microscopy with immunofluorescence staining for the viral E protein showing hemocytes bled from ZIKV infected mosquitoes at 8 days post feeding al low (A) and high magnification (B).

Supplementary figure 1

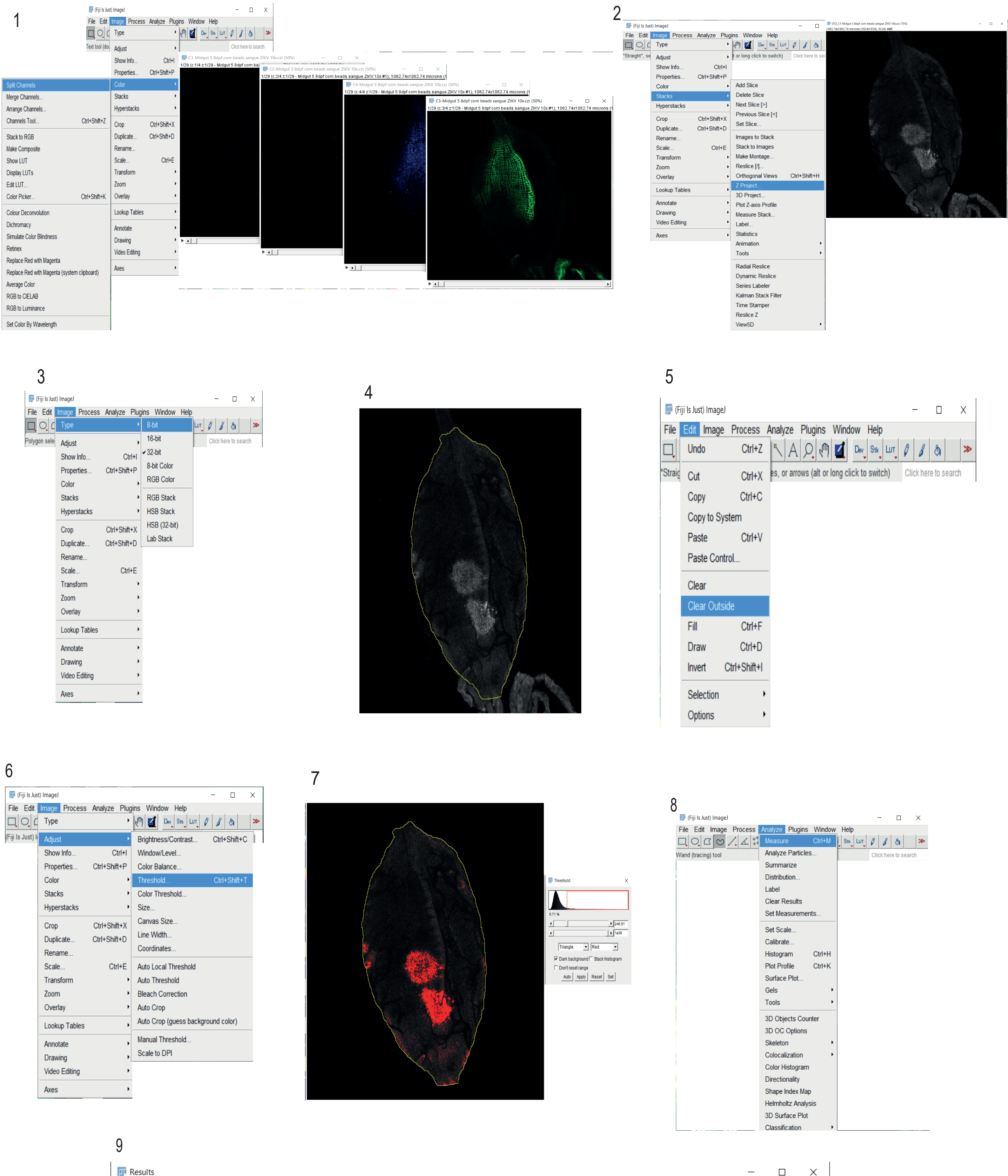

	Label	Area	Mean	Min	Max	IntDen	%Area	RawIntDen	MinThr	MaxThr
1	STD_C1-Midgut 5 8dpf com beads sangue ZIKV 10x.czi	21545.149	79.134	58	255	1704946.911	5.640	1582911	58	255

Supplementary figure 2

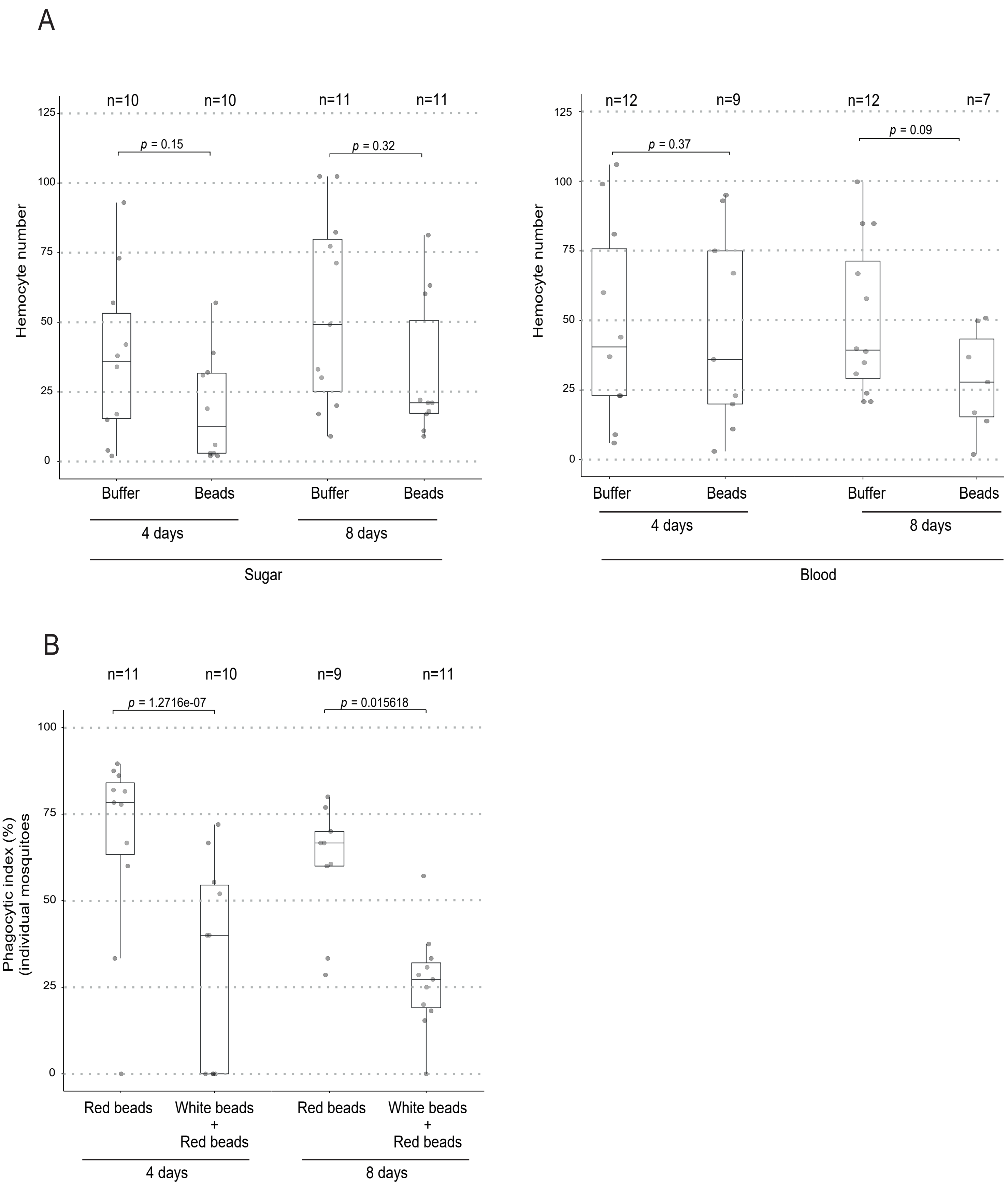

Supplementary figure 3

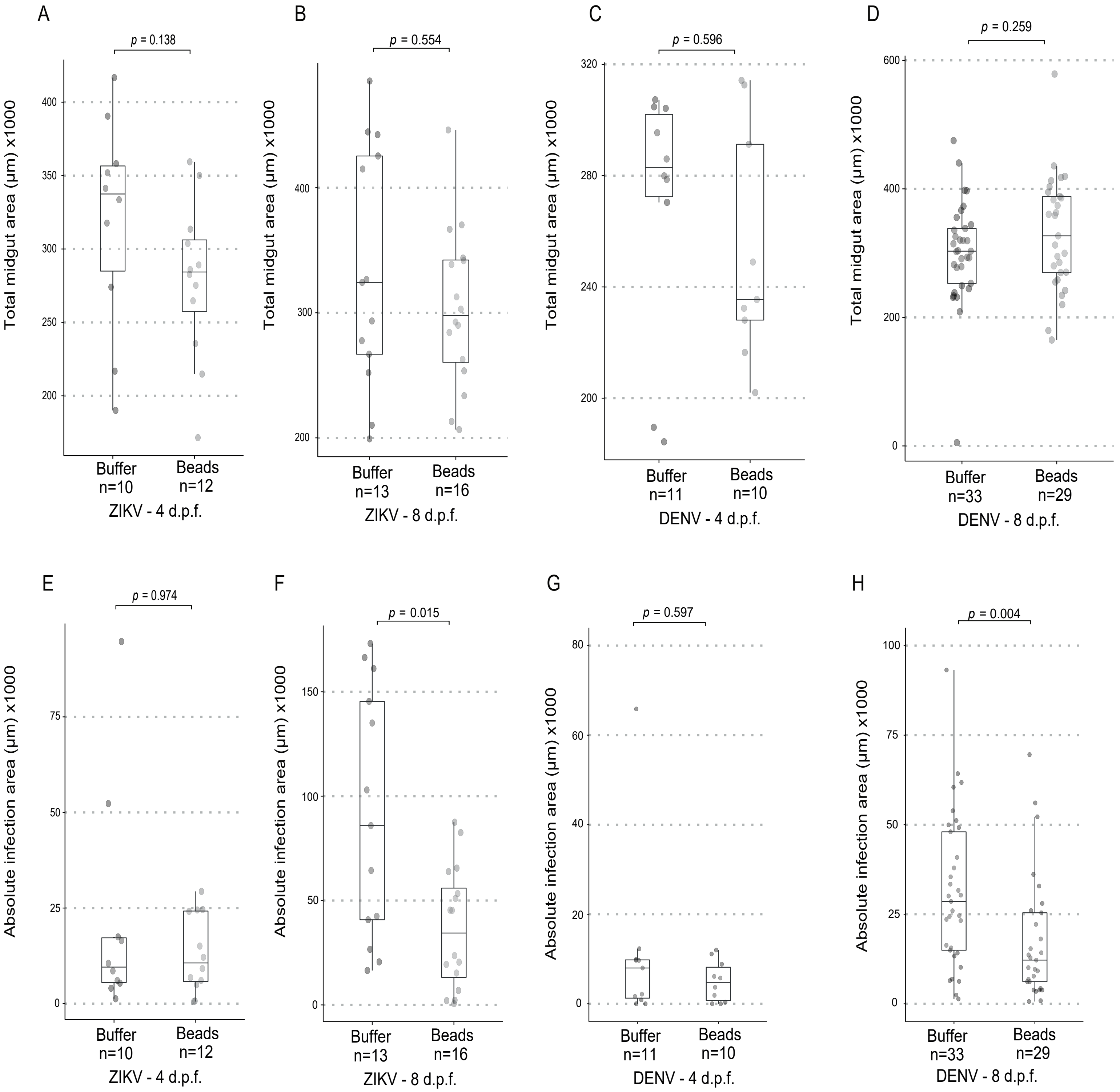

Supplementary figure 4

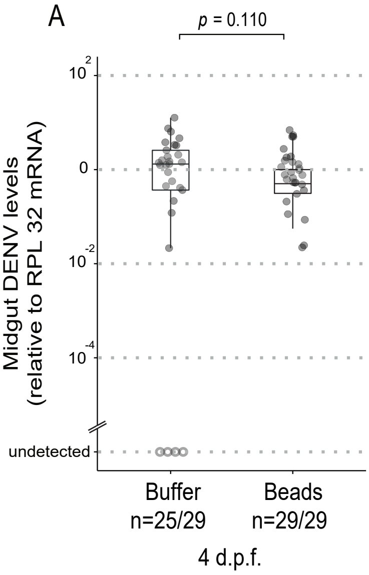

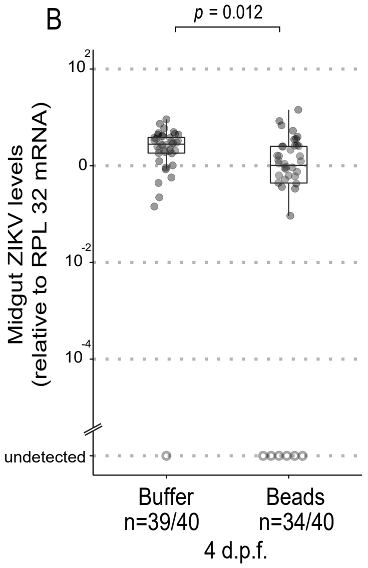

Supplementary figure 5

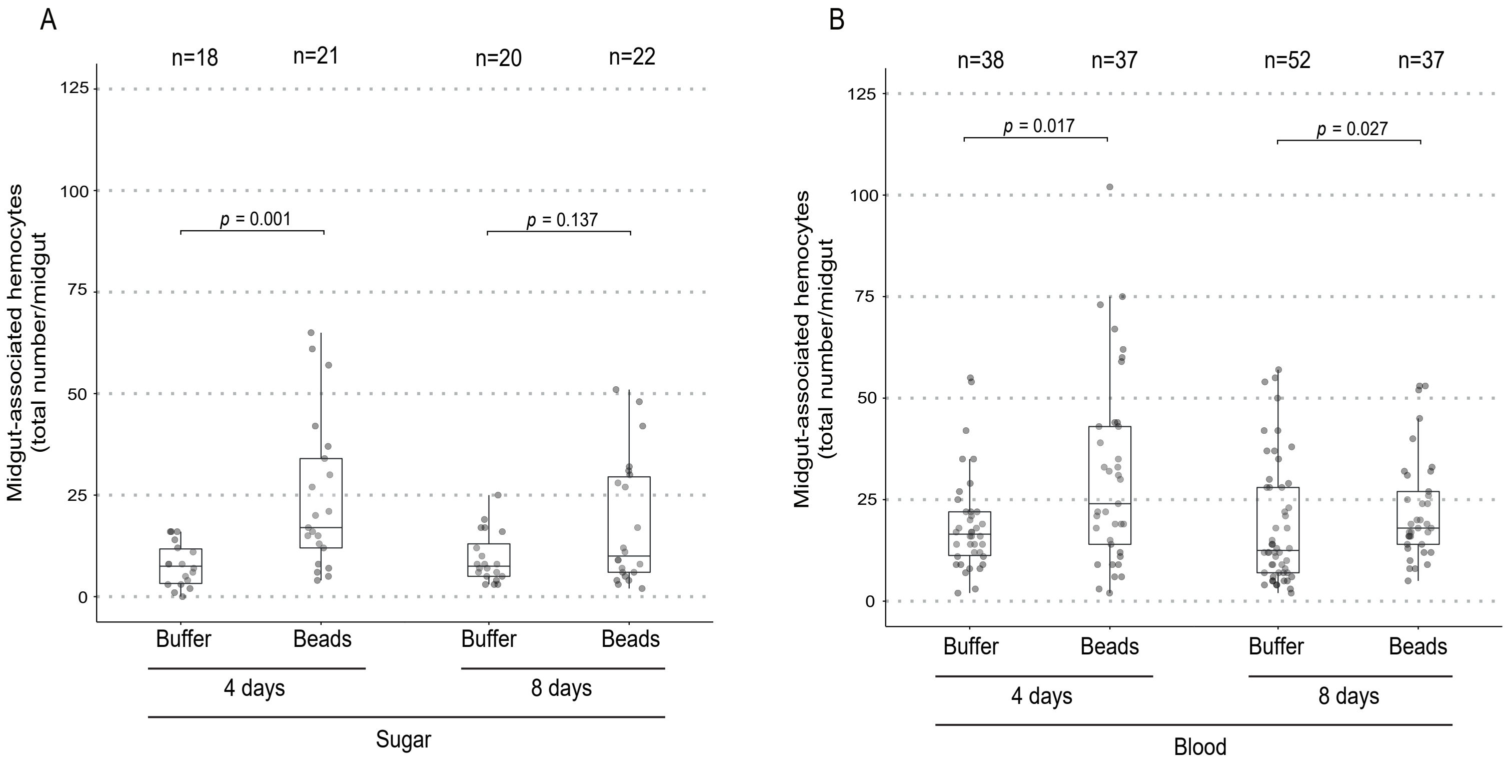

Supplementary figure 6

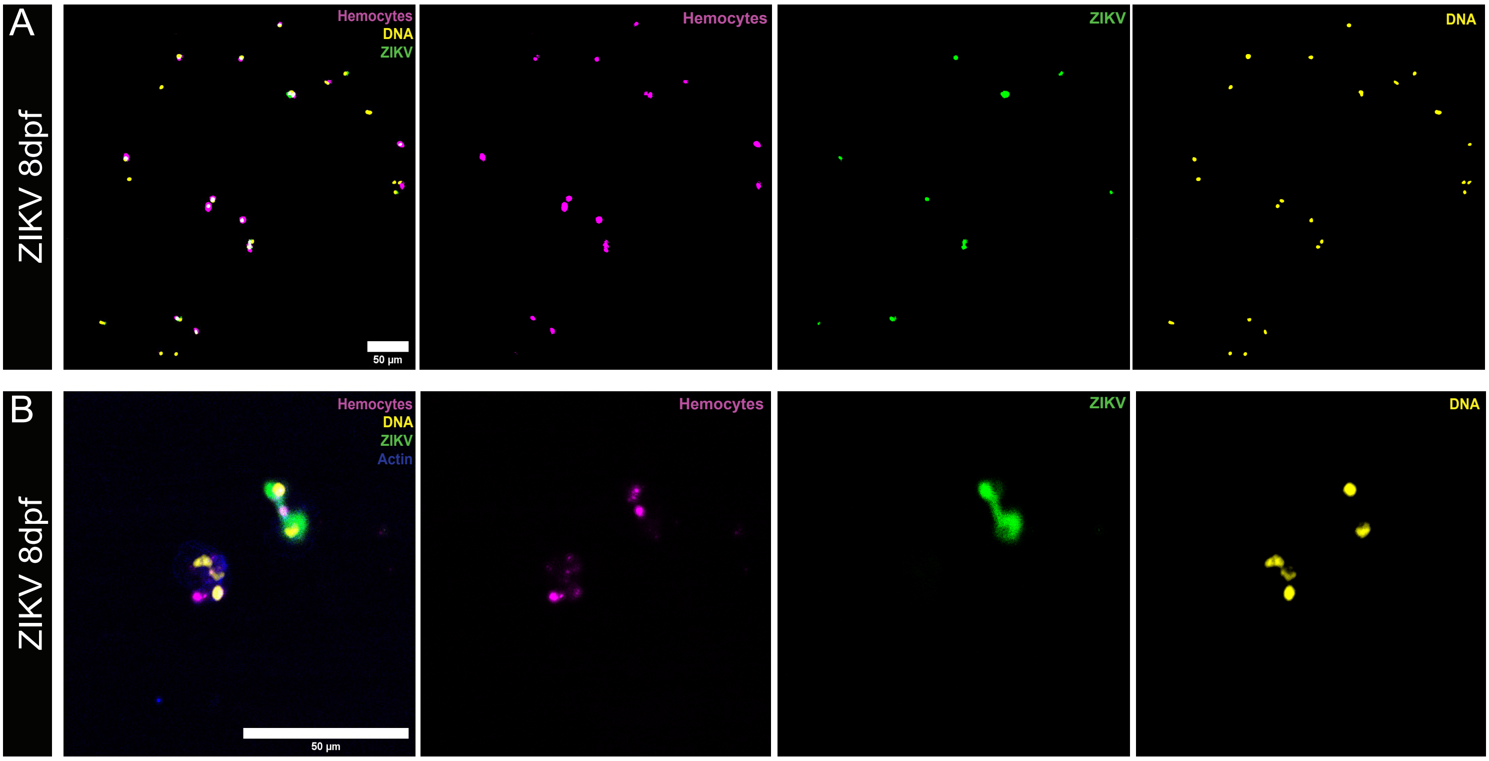

Supplement: Supplementary file 1 [file Presentation_1.pdf]
